# Transcriptome Responses of the Soil-Dwelling Collembolan (*Entomobrya proxima* Folsom) to Fertilizer Type and Concentration

**DOI:** 10.3390/biology13110950

**Published:** 2024-11-19

**Authors:** Xinyue Yang, Gang Li, Weiming Xiu

**Affiliations:** Agro-Environmental Protection Institute, Ministry of Agriculture and Rural Affairs, Tianjin 300191, China; 82101222104@caas.cn

**Keywords:** *Entomobrya proxima* Folsom, transcriptomic responses, fertilizer, DEGs, sustainable agriculture

## Abstract

Soil collembolans have been regarded as the effective bioindicator of environmental changes. However, the physiological mechanisms through which collembolans respond to agricultural activities are largely unknown. Given the plasticity and sensitivity to environmental changes, even subtle responses can be quantified via transcriptomics. Therefore, the relevant in situ soil ecosystem and numerically dominant collembolan species *Entomobrya proxima* Folsom was selected to explore the dynamic responses to fertilizer type and concentration using transcriptome sequencing over three periods (6 h, 24 h and 10 d). The results showed that exposure duration caused significant alterations in gene expression profiles. At day 10 after exposure, gene expression patterns differed remarkably between the two fertilizer types and the control. Relative to organic fertilizer, the number of DEGs was increased by 114.31% under inorganic fertilizer, which declined with increasing inorganic fertilizer concentrations. Functional enrichment analysis was indicative of enhanced fatty acid and carbohydrate metabolism and reduced disease occurrence by organic fertilizer; however, an inhibited lipid synthesis process promoted susceptibility to infection, triggered oxidative stress, etc. by inorganic fertilizer. Overall, fertilizer addition changed the transcriptional pattern of the collembolan, potentially causing shifts in pathways related to metabolism, immunity, etc. In comparison to inorganic fertilizer, organic fertilizer impacted less on the gene expression patterns, implying that organic fertilizer application may be more beneficial to soil animal health.

## Simple Summary

Subtle changes in soil animals may disturb their normal ecological functions and thus affect the homeostasis of soil ecosystems. Transcriptome sequencing technology was used to explore the potential physiological effects of fertilizer application type and concentration on soil collembolan *Entomobrya proxima* Folsom. The results showed that organic fertilizer had less physiological harm to *Entomobrya proxima* Folsom than inorganic fertilizer, which meant that the application of organic fertilizer was more beneficial to the health of soil animals and further realized the sustainable development of agriculture. In addition, efforts should be made to study the effects of long-term fertilization on the functional traits of soil animals in the future, and the transcriptome sequencing technology should be used to identify key genes and their interactions, so as to predict the future functional changes of agricultural ecosystems through the changes of functional traits of soil animals.

## 1. Introduction

Soil faunas are essential to Earth’s life and occupy approximately 23% of the total known soil species [1], which perform a wide array of important ecological functions including decomposition, nutrient cycling, energy transfer and stability maintenance [2]. Collembolan, as one of the ubiquitous and decisive groups of soil faunas, contributes significantly to soil ecosystem composition and function [3,4]. They often appear in areas of high organic matter value where organic particles and microorganisms are ingested [5] and present strong vulnerability to environmental disturbances, which have left them as a model organism and an important bioindicator used in plenty of studies [6,7,8,9]. Most research so far undertaken on collembolans has examined environmental effects where the interest is mainly focused on the fields of soil ecology, ecotoxicology, and ecogenomics related to pollutants [10,11,12]. However, as of yet, the impacts of agricultural practices on the collembolans remain largely unclear.

In the past few decades, inorganic fertilization has always been the major agricultural practice, especially inorganic nitrogen fertilization with the highest application rates of chemical nitrogen fertilizers, which has made the greatest contributions to global food security by influencing cereal yield and fed more than half the population in the world [13]. However, on account of the prevalence of too-intensive agricultural practices represented by inorganic fertilization, detrimental effects were found in relation to the environment and biodiversity, leading to the imbalance of the farmland ecosystem [14,15]. Organic fertilizer application (alone or combined with inorganic fertilizer) has already been proposed as a sustainable agricultural management strategy, which has been increasingly adopted in the current farming systems [16]. Furthermore, organic N fertilization was proven to provide great benefits to the density and biomass of soil animals [17]. It is well-established that any type of fertilizer can significantly and differentially affect soil properties, the diversity of organisms living within the soil, and even their community structure and function [18,19,20,21]. Yet nowadays, the effect mechanisms of organic and inorganic fertilization on soil animals remains largely unexplored, and in particular the response mechanisms of specific soil faunal taxonomic groups with high environmental sensitivity.

Due to the plasticity and sensitivity to environmental changes, even subtle effects can be quantified by transcriptomics [17]. RNA sequencing (RNA-seq) technology is thus used as a powerful high-throughput sequencing to assess environmental effects, and can impartially identify potential regulatory pathways relevant to exogenous stress [22]. A substantial quantity of studies has applied transcriptome analysis for a variety of soil inhabitants following exposure to diverse pollutants, including antibiotics such as polymyxin B [23], and pesticides such as pentachlorophenol [24] and mandipropamid [25]. Moreover, changes in the activities of antioxidant enzymes including catalase (CAT), superoxide dismutase (SOD), etc. can directly reflect the oxidation–antioxidant action in soil fauna [26], which have been utilized to illustrate the direct toxicity from pollutants [27,28]. Hence, the joint application of RNA-seq and enzymatic activity examination can more holistically shed light on the impacts of fertilizer application on soil fauna.

Understanding the soil faunal processes underlying responses to fertilizer addition can lead to more sustainable agricultural management practices to achieve a win–win situation. For this purpose, *Entomobrya proxima* Folsom, an in situ soil ecosystem relevant and numerically dominant epiedaphic collembolan species derived from the farmland in North China and with increasing attention in recent years [29,30], was selected as the research object, and meanwhile, an indoor exposure experiment was performed in the current study. It is anticipated that the findings will provide an initial assessment of transcriptional and physiological responses of collembolans to fertilizer exposure and give a comprehensive view of the effects of exogenous fertilizer on soil fauna.

## 2. Materials and Methods

### 2.1. Preparation of Collembolans and Feed

*Entomobrya proxima* Folsom (Collembola: Entomobryidae) (abbreviated as *E. proxima* below) used in the experiment were originally isolated from the cropland at Wuqing Experiment Station for Field Observation of Farmland Ecosystem, Chinese Academy of Agricultural Sciences (Tianjin, China) (117°12′ E, 39°21′ N), where the climate type is classified as a temperate continental monsoon climate, exhibiting a mean annual temperature of 12.9 °C and mean annual precipitation of 523.0 mm. The test collembolans were cultivated for more than 1 year in the laboratory. According to the standardized methods of the Organization for Economic Co-operation and Development (OECD, Paris) [31], moist plaster and charcoal were mixed (at the ratio 8:1 in *w*/*w*) in Petri dishes (75 mm), which were used as the culture containers for collembolans to provide a suitable growth environment. The collembolans were precultured for one week in an incubator at a temperature of 21 ± 2 °C, 75% humidity and 16:8 h (dark:light, 800lx) to acclimatize them to the new environment [32]. Yeast (Angel) provided as feed and ultrapure water were added to the Petri dishes every 6 days and every 3 days, respectively.

### 2.2. Design of Experiment

The experiment was carried out in a manner of dietary exposure, which was adopted to simulate collembolan feeding in the scenario of agricultural organic and inorganic fertilization. Two experimental groups, organic group and inorganic group (named group O and group I, respectively) were established, and each group included three treatments with six replicates. The three treatments within organic group were as follows: feed containing 1% of organic fertilizer (treatment O1), feed containing 6% of organic fertilizer (treatment O2), and feed containing 10% of organic fertilizer (treatment O3). The inorganic group consisted of the following three treatments: feed mixed with 1% inorganic fertilizer (treatment I1), feed mixed with 4% inorganic fertilizer (treatment I2), and feed mixed with 6% inorganic fertilizer (treatment I3). A serial of homogenized mixtures of yeast, specific ratios of organic or inorganic fertilizer as described above, and ultrapure water were initially made as the feed. At the same time, a control with only yeast was set (treatment CK), and also replicated six times. A total of 42 dishes were obtained after multiplying seven (treatments) by six (dishes, i.e., replicates). The feed used in treatment CK was made of yeast and ultrapure water.

For all kinds of feed, after being frozen at −80 °C, the mixtures were transferred into a freeze dryer to remove moisture before use. Subsequently, the mixtures were ground thoroughly and stored at −80 °C for subsequent use. The organic fertilizer tested in this study was manufactured by Lianhua Health Industry Group Co., Ltd. (Xiangcheng, Henan province, China) with the following characteristics: ≥45% organic matter and ≥ 5% other elements (N + P_2_O_5_ + K_2_O), and urea (CH_4_N_2_O) was chosen as the representative of inorganic nitrogen fertilizer due to the prevalence and highest application rate.

At the start of experiment, one hundred *E. proxima* individuals, starved for 48 h and aged at 13–15 d, were transferred into each Petri dish and fed with 0.1 g of the corresponding feed described above. The culture conditions throughout the whole experimental period were set as described above, and feed and sterile water were renewed on schedule.

### 2.3. Sample Collection

In the current experiment, the collembolan samples were, respectively, gathered after exposure for 6 h, 24 h and 10 d (named period A, period B and period C, respectively). At each sampling period, 50 *E. proxima* individuals from two dishes of the same treatment were pooled and placed in 1.5 mL centrifuge tubes in order to generate a composite sample to provide a sufficient amount of RNA, which was quickly transferred into liquid nitrogen. As such, three replicates were reacquired for each treatment. After thoroughly frozen, the samples were stored at −80 °C before RNA isolation. Eventually, a total of 63 samples for RNA-seq analysis were obtained.

### 2.4. Transcriptome Analysis

To identify the potential regulation pathways of collembolans induced by exogenous fertilizer application, eukaryotic transcriptome sequencing was undertaken. Total RNA was extracted from each sample using TRIzol^®^ Reagent. RNA purification, reverse transcription, library construction and sequencing were performed by Shanghai Majorbio Bio-pharm Biotechnology Co., Ltd. (Shanghai, China) using corresponding kits according to manufacturers’ instructions. Subsequently, the expression levels of each processing gene and differentially expressed genes (DEGs) were determined. The DEGs were identified by setting a *p* ≤ 0.05 and an absolute value of log_2_Radio ≥ 1. Enrichment analysis was used to identify the biological functions of DEGs, and determine the biological processes, functions or diseases which were intimately associated with significantly expressed gene sets as induced by fertilizer addition. Therefore, the identified DEGs were divided into upregulated and downregulated DEGs, and mapped to terms using the Gene Ontology (GO) and Kyoto Encyclopedia of Gene and Genomes (KEGG) to determine the corresponding pathways. The top 20 of both GO terms and KEGG pathways with significant enrichment in all fertilizer treatments were presented via bubble chart [23].

### 2.5. Measurement of Antioxidant Enzyme Activities

Catalase (CAT) can protect cells from oxidative damage via decomposition of hydrogen peroxide into water and oxygen [33], and Superoxide dismutase (SOD) is able to clear the excessive amounts of oxygen free radicals to protect the organism against oxidative damage [34,35]. In the present study, activities of CAT and SOD of collembolans were determined to confirm the generation of oxidative stress using Catalase (CAT) Activity Assay Kit and Superoxide Dismutase (SOD) Activity Assay Kit (Solarbio, Beijing, China), respectively, following the instructions of manufacturer. Sampling for measurement of enzymatic activities was also performed at three periods in the same manner as above. At each sampling period, except for the individuals collected for transcriptome analysis, another 6 individuals were randomly gathered and placed into a 1.5 mL centrifuge tube, which were washed using a 2.5% sodium hypochlorite solution for three times and rinsed with sterile water for five times [32]. The enzymatic activities were expressed as U per individual.

### 2.6. Statistical Analysis

Principal Coordinated Analysis based on the Bray–Curtis distance [36] with Adonis test was conducted to evaluate whether the differences in gene expression patterns between groups, as well as treatments were significant with vegan 2.6.4 in R version 4.2.3. The Volcano map illustrating DEGs between all treatments with inorganic fertilizer and treatment CK at each sampling period was plotted using tidyverse 2.0.0 and ggplot2 3.5.0 in R version 4.2.3. The statistical analysis of CAT and SOD activities was performed using EasyStat 0.1.0 in R version 4.2.3, with all data presented as mean ± error deviation (SE). One-way analysis of covariance (one-way ANOVA) was also conducted with package EasyStat to assess the significance of differences between treatments at a significance level of *p* = 0.05 and the Duncan HSD test was utilized for multiple comparisons. The correlation between enzymatic activities and gene expression level for DEGs in inorganic fertilizer treatments across three periods was performed through Prism software (https://www.graphpad.com/updates (accessed on 14 November 2024)) based on Spearman rank correlation coefficient. Two-way analysis of variance was used to investigate the effects of exposure duration and exposure concentration of inorganic fertilizer on the number of DEGs, expression of common DEGs from pathways of glutathione metabolism and arachidonic acid metabolism, as well as CAT and SOD activities through tidyverse 2.0.0 and ggpolt2 3.5.0 in R version 4.2.3.

## 3. Results

### 3.1. Overview of RNA-Seq

A total of 446.59 Gb Clean Data were obtained, with above 6.02 Gb Clean Data for each sample. The percentage of Q30 bases was over 91.78%. Based on the transcriptome research without a reference genome, all high-quality RNA-seq reads obtained after quality control were generated by de novo assembly to generate contigs and single sequences. The sequencing data were submitted to NCBI under Bio Project PRJNA1183896.

### 3.2. Effects of Fertilizer Exposure Duration, Type and Concentration on Gene Expression Pattern

**Figure 1:**
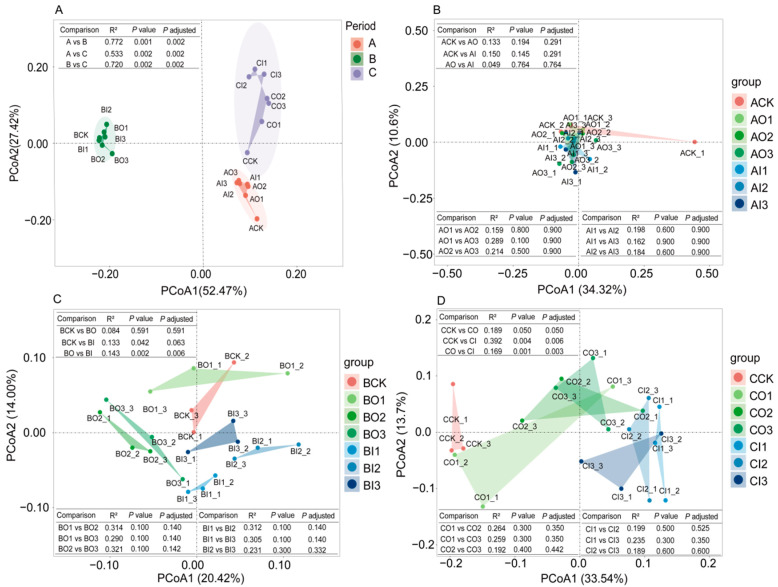
PCoA plots of the effects of exposure duration, type and concentration of fertilizer on gene expression profile of *E. proxima*. (**A**) PCoA plots of the effects of all exposure duration of fertilizer on gene expression profile of *E. proxima*. (**B**) PCoA plots of the effects of exposure type and concentration of fertilizer on gene expression profile of *E. proxima* in period A. (**C**) PCoA plots of the effects of exposure type and concentration of fertilizer on gene expression profile of *E. proxima* in period B. (**D**) PCoA plots of the effects of exposure type and concentration of fertilizer on gene expression profile of *E. proxima* in period C. Note: A: period A, 6 h exposure duration, B: period B, 24 h exposure duration, C: period C, 10 d exposure duration, O1: feed mixed with 1% organic fertilizer, O2: feed mixed with 6% organic fertilizer, O3: feed mixed with 10% organic fertilizer, I1: feed mixed with 1% inorganic fertilizer, I2: feed mixed with 4% inorganic fertilizer, I3: feed mixed with 6% inorganic fertilizer. CK: feed with only yeast.

PCoA analysis with the Adonis test was here performed to illustrate the influences of exposure duration, type and concentration of fertilizer. Samples collected at the same sampling period were observed to cluster together (periods A, B and C), indicating a significant influence of exposure duration on gene expression (*p* adjust = 0.002) (Figure 1A). Furthermore, regardless of treatments, all samples at period A gathered together with only one sample (named ACK_1) showing obvious separation from other samples (Figure 1B). And at period B, all treatments showed separation from each other with an overlap solely observed between treatment O1 and treatment CK, and both type and concentration of organic and inorganic fertilizer had no significant impacts on the gene expression pattern of *E. proxima*. (Figure 1C). After exposure for 10 days (at period C), the treatments within group O were congregated but separated from treatment CK, which was true for the treatments belonging to group I. There were remarkable influences on collembolan gene expression profile by fertilizer type compared to the control (*p* adjust = 0.050 for organic fertilizer and *p* adjust = 0.006 for inorganic fertilizer) (Figure 1D). The gene expression profile between group O and group I had no significant differences at period A, which yet differed markedly at periods B and C (*p* adjust = 0.006 at period B and *p* adjust = 0.003 at period C).

### 3.3. Effects of Fertilizer Type on DEGs

#### 3.3.1. DEGs Analysis

**Figure 2:**
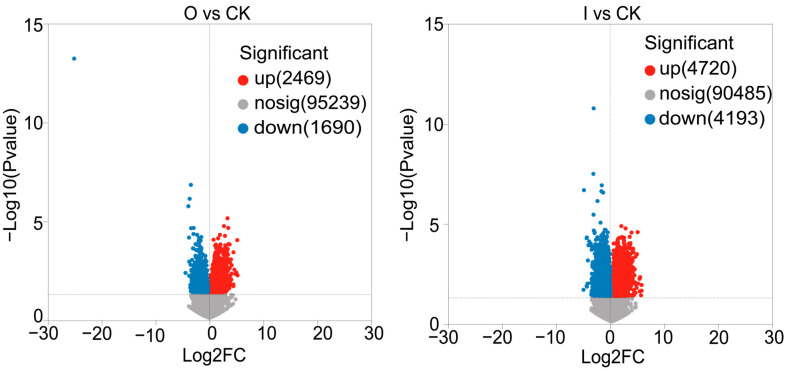
Volcano maps of DEGs with comparison between groups O and I. Note: FC: fold change, O: all organic fertilizer treatments over three periods, I: all inorganic fertilizer treatments over three periods, CK: all CK treatments over three periods.

The number of DEGs in inorganic fertilizer treatments was higher than that in organic fertilizer treatments. Organic fertilizer exposure generated 4159 DEGs, of which 1690 genes were downregulated and 2460 genes upregulated. Inorganic fertilizer exposure resulted in more DEGs, the number was up to 8913 with 4720 upregulated and 4193 downregulated genes, respectively (Figure 2).

#### 3.3.2. GO and KEGG Enrichment Analysis

**Figure 3:**
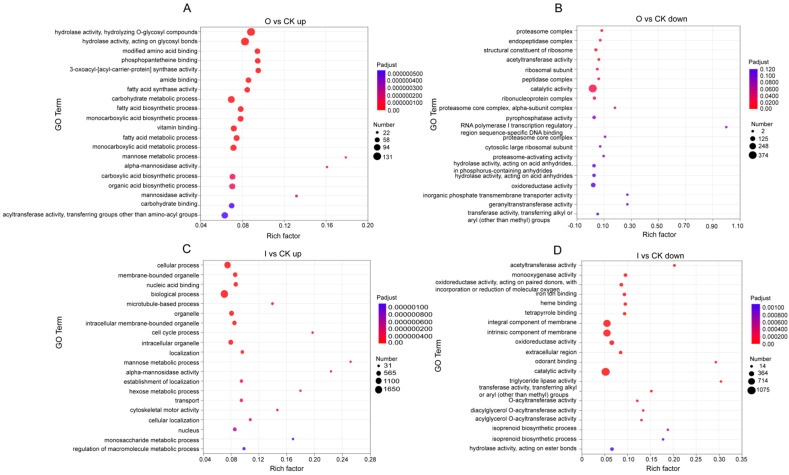
Bubble charts of up and downregulated genes in organic and inorganic treatments compared to the control, which were presented with top 20 GO pathways enriched by DEGs. Note: (**A**) Bubble chart of the enriched GO pathways of the upregulated DEGs in treatment O. (**B**) Bubble chart of the enriched GO pathways of the downregulated DEGs in treatment O. (**C**) Bubble chart of the enriched GO pathways of the upregulated DEGs in treatment I. (**D**) Bubble chart of the enriched GO pathways of the downregulated DEGs in treatment I.

GO analysis assigned DEGs into three major categories, including molecular function (MF), cellular component (CC) and biological process (BP). Among the functional GO terms, mannose metabolic process (BP), alpha-mannosidase activity (MF) and mannosidase activity (MF) were most enriched with upregulated DEGs under organic treatment (Figure 3A), whereas proteasome complex (CC), endopeptidase complex (CC) and acetyltransferase activity (MF) were most enriched with downregulated DEGs (Figure 3B). Under inorganic treatment, the terms most enriched with upregulated DEGs were mannose metabolic process (BP), alpha-mannosidase activity (MF) and cell cycle process (BP) (Figure 3C). Contrastingly, triglyceride lipase activity (MF), odorant binding (MF) and acetyltransferase activity (MF) were most enriched with downregulated DEGs (Figure 3D).

**Figure 4:**
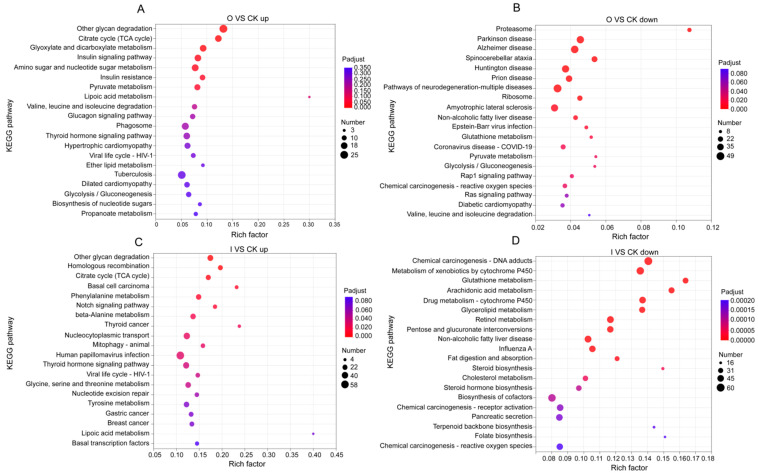
Bubble charts of up and downregulated genes in organic and inorganic treatments compared to the control, which were presented with top 20 KEGG pathways enriched by DEGs. Note: (**A**) Bubble chart of the enriched KEGG pathways of the upregulated DEGs in treatment O. (**B**) Bubble chart of the enriched KEGG pathways of the downregulated DEGs in treatment O. (**C**) Bubble chart of the enriched KEGG pathways of the upregulated DEGs in treatment I. (**D**) Bubble chart of the enriched KEGG pathways of the downregulated DEGs in treatment I.

According to KEGG, these pathways were broadly categorized into Human Disease (H), Cellular Processes (C), Metabolism (M), Organismal Systems (O), Environmental Information Processing (E) and Genetic Information Process (G). KEGG analysis showed that pathways like other glycan degradation (M), citrate cycle (TCA cycle) (M) and glyoxylate and dicarboxylate metabolism (M) were the most enriched among the upregulated DEGs, yet pathways such as proteasome (G) and multiple human diseases (H) were enriched among the downregulated DEGs under organic treatment (Figure 4A,B). Under inorganic treatment, pathways affiliated to thyroid cancer (H), basal cell carcinoma (H) and homologous recombination (G) were the most enriched among the upregulated DEGs, while glutathione metabolism (M), arachidonic acid metabolism (M) and steroid biosynthesis (M) pathways were enriched among the downregulated DEGs (Figure 4C,D).

### 3.4. Changes in Antioxidant Enzymatic Activities Under Inorganic Fertilizer Treatment and Correlations with Common Genes

**Figure 5:**
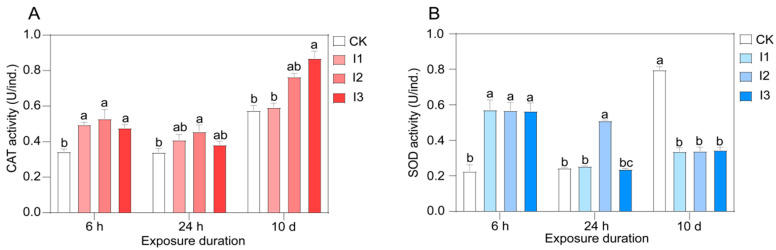
Antioxidant enzymatic activities of *E. proxima* under inorganic fertilizer treatment. Note: (**A**) CAT activity, (**B**) SOD activity, I1: feed mixed with 1% inorganic fertilizer, I2: feed mixed with 4% inorganic fertilizer, I3: feed mixed with 6% inorganic fertilizer. CK: feed with only yeast. Different lowercase letters indicate significant differences in enzyme activities between different treatments at the same exposure time.

The results showed that CAT activity was significantly increased by inorganic fertilizer addition at periods A compared with the control (*p* < 0.05), with the highest activity observed in treatment I2, followed by treatments I1 and I3, and there was no significant difference between the treatments with inorganic input. Similarly, CAT activity was higher in treatments with inorganic input than in treatment CK; however, a considerable change was observed only for treatment I2 (*p* < 0.05). A tendency of continuous rise was found at period C, and remarkably higher activity was observed at the highest concentration compared to the control (*p* < 0.05) (Figure 5A). With respect to SOD activity, inorganic fertilizer input also caused a significant decrease compared to the control following exposure for 6 h (*p* < 0.05), and no obvious changes were found between treatments with inorganic fertilizer. However, in sharp contrast at period C, the SOD activity was significantly lowered by inorganic fertilizer addition in comparison with the control (*p* < 0.05). And at period B, treatment I2 was found to have the highest activity with remarkable divergence from the other treatments (*p* < 0.05) (Figure 5B). The activities were a little higher and lower under treatments I1 and I3 than under treatment CK, respectively (Figure 5B).

**Figure 6:**
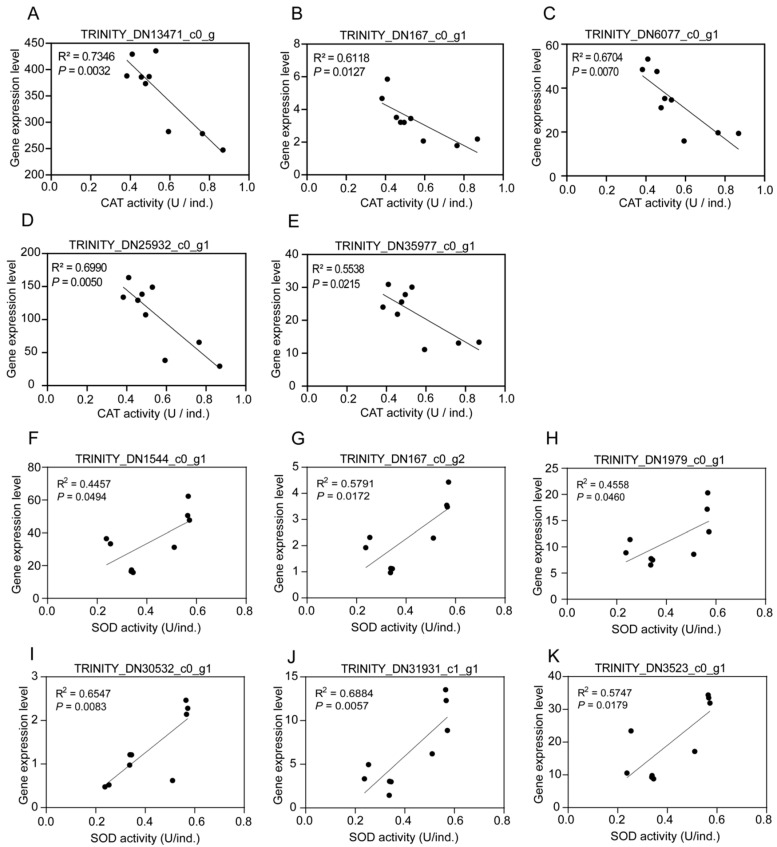
Correlation analysis of gene expression level with antioxidant enzymatic activities. Note: (**A**–**E**) represent correlation analyses between CAT activity and expression level of five genes selected, respectively, and (**F**–**K**) represent correlation analyses between SOD activity and expression level of six genes selected, respectively.

In this study, the commonly enriched genes categorized into glutathione metabolism (M) and arachidonic acid metabolism (M) pathways which are thought to be closely linked to oxidative stress, were selected to analyze the correlations between gene expression level and antioxidant enzyme activities. Five common genes (TRINITY_DN13471_c0_g2, TRINITY_DN167_c0_g1, TRINITY_DN25932_c0_g1, TRINITY_DN35977_c0_g1 and TRINITY_DN6077_c0_g1) were further chosen for correlation analysis with CAT enzyme activity. It was found that the expression level of the above five genes was significantly negatively correlated with the CAT enzyme activity (Figure 6A–E). Furthermore, six common genes (TRINITY_DN1544_c0_g1, TRINITY_DN167_c0_g2, TRINITY_DN1979_c0_g1, TRINITY_DN30532_c0_g1, TRINITY_DN31931_c1_g1 and TRINITY_DN3523_c0_g1) were selected to illustrate the relationship with SOD activity. Contrary to the result of CAT, the findings indicated a significantly positive correlation of SOD activity with the gene expression level (Figure 6F–K). In addition, the results of a two-factor analysis of variance showed that the expression of all the above DEGs was significantly affected by inorganic fertilizer exposure duration, and only TRINITY_DN1979_c0_g1 and TRINITY_DN25932_c0_g1 were significantly affected by inorganic fertilizer exposure duration and concentration, as seen in Table 1. The activities of CAT and SOD enzymes were significantly influenced by exposure duration, concentration of inorganic fertilizer and the two factors together, as seen in Table 2.

### 3.5. Effects of Inorganic Fertilizer Concentrations on DEGs

#### 3.5.1. DEGs Analysis

**Figure 7:**
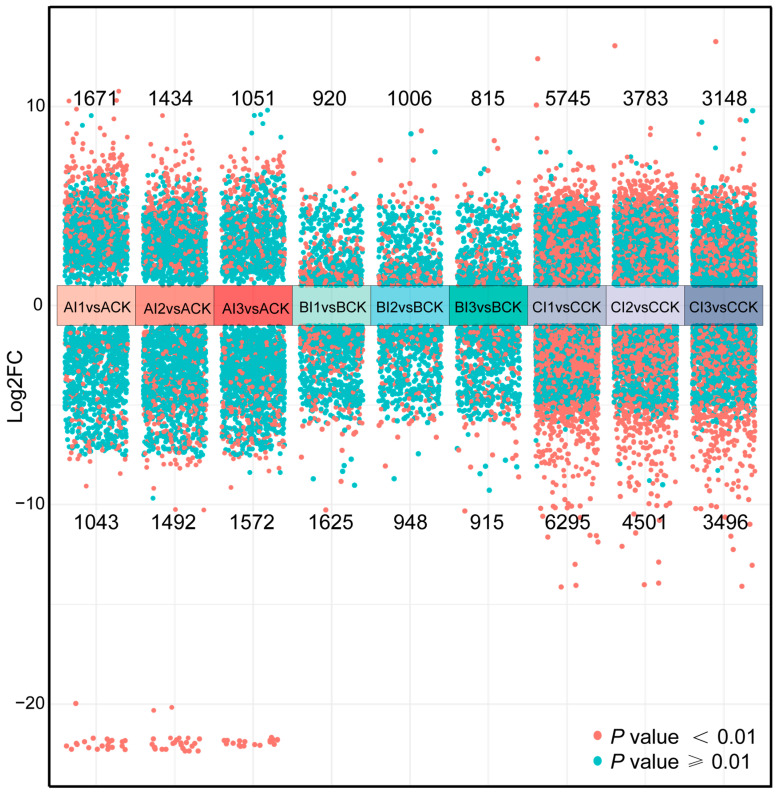
Volcano map of differentially expressed genes (DEGs) under all treatments added with inorganic fertilizer over three periods. Note: A: period A, 6 h exposure duration, B: period B, 24 h exposure duration, C: period C, 10 d exposure duration, I1: feed mixed with 1% inorganic fertilizer, I2: feed mixed with 4% inorganic fertilizer, I3: feed mixed with 6% inorganic fertilizer. CK: feed with only yeast.

Since inorganic fertilizer treatments generated more DEGs than organic fertilizer treatments and caused intense oxidative stress, further analyses of fertilizer concentration effects on *E. proxima* were conducted for treatments added with inorganic fertilizer. As seen in Figure 7, more DEGs were found at low concentrations with a tendency to decrease with increasing concentrations. However, an exception was observed in period A in that the number of downregulated DEGs increased with increasing concentrations. The results of a two-way analysis of variance of exposure duration and concentration of inorganic fertilizer showed that exposure duration was the main factor driving the change in the number of DEGs, as seen in Table 3.

#### 3.5.2. GO Enrichment Analysis

**Figure 8:**
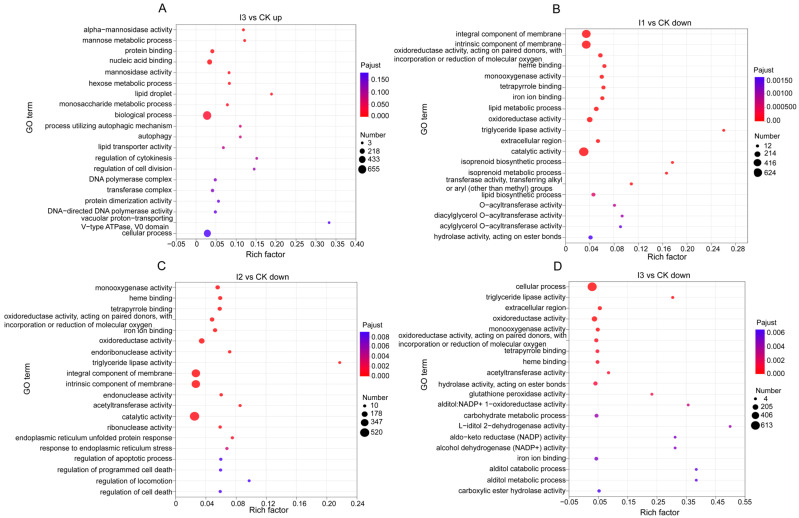
Bubble charts of up and downregulated genes in different concentrations in inorganic treatments with different concentrations, which were presented with top 20 GO pathways enriched by DEGs. Note: (**A**) Bubble chart of the enriched GO pathways of the upregulated DEGs in I3 treatments I3. (**B**) Bubble chart of the enriched GO pathways of the downregulated DEGs in I1 treatments I1. (**C**) Bubble chart of the enriched GO pathways of the upregulated DEGs in I2 treatments I2. (**D**) Bubble chart of the enriched GO pathways of the downregulated DEGs in I3 treatments I3.

The functional GO terms including lipid droplet (CC), mannose metabolic process (BP) and alpha-mannosidase activity (MF) were most enriched under treatment I3 as indicated with upregulated DEGs (Figure 8A). Terms like triglyceride lipase activity (MF), isoprenoid biosynthetic and metabolic process (BP) were most enriched under treatment I1 with DEGs downregulated. The regulation of apoptotic process and cell death (BP) appeared in the top 20 categories under treatment I2 (Figure 8B,C). In treatment I3, downregulated DEGs were enriched in L-iditol 2-dehydrogenase activity, NADP activity, NADP+ activity and other multiple enzyme activity terms (Figure 8D). The enrichment of DEGs under other treatments was not significant.

#### 3.5.3. KEGG Enrichment Analysis

**Figure 9:**
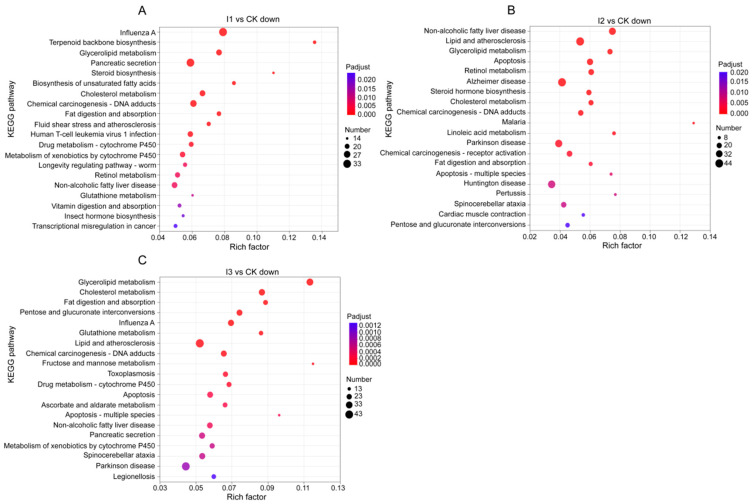
Bubble chart of up and downregulated genes in three inorganic treatments compared to the control, which were presented with top 20 KEGG pathways enriched by DEGs. Note: (**A**): Bubble chart of the enriched KEGG pathways of the downregulated DEGs in treatment I1. (**B**): Bubble chart of the enriched KEGG pathways of the downregulated DEGs in treatment I2. (**C**): Bubble chart of the enriched KEGG pathways of the downregulated DEGs in treatment I3.

KEGG analysis showed that pathways involving terpenoid backbone biosynthesis (M), steroid biosynthesis (M) and biosynthesis of unsaturated fatty acids (M) were the most enriched among the downregulated DEGs in treatment I1 (Figure 9A). In treatment I2, pathways encompassing malaria (H), linoleic acid metabolism (M) and non-alcoholic fatty liver disease (H) were the most enriched among the downregulated DEGs, and pathway apoptosis (C) enriched were also discovered (Figure 9B). Pathways such as glycerolipid metabolism (M), fructose and mannose metabolism (M) and apoptosis-multiple species (C) were the most enriched among the downregulated DEGs in treatment I3 (Figure 9C). The enrichment of DEGs under other treatments was not significant.

## 4. Discussion

### 4.1. Effects of Fertilizer Exposure Duration, Type and Concentration on Gene Expression of E. proxima

Overall, fertilizer exposure duration imposed a significant impact on the gene expression of *E. proxima*, which was similar to the finding that sampling periods linked with individual developmental stages drove the significant changes in the gene expression of an echinoderm [36]. With respect to the gene expression pattern at each period, obvious divergences were observed. At the period of exposure for 6 h, the close agglomeration of the samples indicates a similar expression pattern of all treatments, suggesting no remarkable variations occurred at the early exposure stage. As the exposure process progressed, apparent separation among treatments emerged in accompanying exposure for 24 h, albeit for an overlap between treatments CK and O1, which intimates relatively more similar patterns at low organic fertilizer with the control and thus fewer impacts in this scenario. In the meantime, expression patterns differed significantly between groups O and I, suggesting contrasting effects by fertilizer types. It is believed that the genes of an organism will be stably expressed after reception of a relatively long stimulation, resulting in a stationary expression profile. After 10 days of exposure, there were significant differences in the expression pattern between group O, group I and treatment CK, which was corroborated both by obviously separate clusters of respective samples and adjusted *p*-values via Adonis test. Moreover, the treatments within group O were more adjacent to treatment CK, revealing more similar gene expression patterns and fewer influences caused by external input, in particular the treatment with low organic fertilizer concentration (partially like the above-described result at period B), despite the remarkable discrepancy between group O and treatment CK. It could be, therefore, inferred that the tested collembolan species (*Entomobrya proxima* Folsom) utilized distinct transcriptional strategies to respond to organic and inorganic fertilizer [37], and the overall impacts might be relatively smaller in the context of organic fertilizer input compared to inorganic fertilizer input, insinuating potentially greater alterations in physiology and epigenetic features of collembolans treated with inorganic fertilizer. Moreover, although the experiment ensured the consistency of the age of the *E. proxima*, it could not distinguish the gender of the *E. proxima*, and there were differences between individuals, so the overlap between biological relevance was small.

### 4.2. Effects of Fertilizer Type on DEGs and Antioxidant Enzymatic Activities of E. proxima

The number of DEGs caused by inorganic fertilizer was 114.31% higher than that by organic fertilizer, which meant that organic fertilizer had less influence on gene expression and physiological function of *E. proxima* than inorganic fertilizer. Both organic and inorganic fertilizers reduced acetyltransferase activity and enhanced mannose metabolism. Acetyltransferase is essential for chromatin-based biological processes, including chromatin replication, DNA damage repair, and transcriptional regulation [38], which means that fertilizer addition may cause reproductive and genetic problems of *E. proxima*, leading to changes in the evolution direction of the whole community of collembolans. Mannose metabolism can restore intestinal homeostasis by blocking TNF-α-mediated pro-inflammatory pathways and inhibiting pathological endoplasmic reticulum (ER) stress through the normalization of protein N-glycosylation [39]. It is reasonable to believe that both fertilizers may lead to abnormalities in chromatin-related activities and inflammatory responses in collembolans. Therefore, it was speculated that fertilizer application may cause a large number of misfolded proteins to be preserved in the ER, resulting in ER stress and intestinal inflammation of *E. proxima*. Mannose metabolism is activated as a pathway to inhibit this stress. Additionally, organic fertilizer promoted metabolic processes including polysaccharide degradation, citric acid cycle, as well as aldehyde and dicarboxylate metabolism and metabolism improvement that may promote immunity enhancement, which could explain the enrichment of downregulated disease-related genes. Furthermore, the enrichment of downregulated genes relevant to proteasomes and peptidases suggests that organic fertilizer may impact various protein-related life activities [40], which will affect the normal ecological function of the collembolans in the food web, and thus the soil health. Inorganic fertilizer led to multiple lipid metabolism abnormalities and enriched upregulated genes related to multiple diseases. At the same time, inorganic fertilizer promoted homologous recombination processes and accelerated cell cycles, but excessive homologous recombination can lead to genomic instability which may explain the phenomenon of enrichment of upregulated genes associated with various diseases [41]. In general, fertilizer application can cause changes in the physiological function of *E. proxima*, but the effect of organic fertilizer was more moderate, implying that short-term organic fertilizer application may have less physiological impacts on soil animals, which may further reduce the disturbance of long-term organic fertilizer application on the evolution direction of functional traits of soil animals, so as to maintain the role of soil animals in the ecosystem. Overall, organic fertilizer application was more conducive to the stability of the agricultural ecosystem and the realization of sustainable agricultural development than inorganic fertilizer application.

The CAT activity was significantly increased owing to inorganic fertilizer addition over three periods compared with the control, which was consistent with the results of Zhang [42] that enhanced CAT activity was observed after oxidative stress. In comparison to the control, a significant increase in the activity of SOD because of the addition of inorganic fertilizer was found in all inorganic treatments at period A, which was also true for treatment I2 at period B; however, a sharply contrasting change was found at period C, and all inorganic treatments showed a substantial decline in SOD activity. A similar finding by Hu [43] indicated an overall trend of first increasing and then decreasing under environmental stress conditions in treatments versus control. The oxidative stress caused by the addition of exogenous inorganic fertilizer implied that there may be some resistance mechanism to maintain its homeostasis by regulating enzyme activity. Moreover, a previous study illustrated that downregulated genes affiliated with glutathione and arachidonic acid were enriched [44], which was proven to be induced by intense oxidative stress, and such a finding was corroborated in the current study. Moreover, there were significant correlations between the expression levels of screened genes categorized into pathways glutathione metabolism (M) and arachidonic acid metabolism (M) and the activities of antioxidant enzymes under inorganic fertilizer (negative correlation for CAT yet positive for SOD). In view of this, it could be speculated that inorganic fertilizer is able to cause oxidative stress of *E. proxima*. The activities of CAT and SOD enzymes were affected by both the exposure duration and concentration of inorganic fertilizer. It was speculated that long-term and abundant N sources might induce a large amount of reactive oxygen species, and the generation of antioxidant enzymes also resulted in the accumulation of reactive oxygen species due to the above physiological function obstacles, which further affected the normal physiological function. The formed positive feedback mechanism may lead to physiological disorders of *E. proxima*.

### 4.3. Effects of Inorganic Fertilizer Concentration on DEGs of E. proxima

Inorganic fertilizer at low addition concentration caused most DEGs after exposure 10 d. Since urea was selected as the inorganic fertilizer in this experiment, it was speculated that N content is the main cause of the appearance of differential genes, that is, long-term low N content tended to change (upregulate or downregulate) the expression of more genes. The downregulated genes were mainly enriched in the lipid synthesis process, suggesting that the low inorganic fertilizer input hindered the lipid synthesis process. With the increase in inorganic fertilizer concentration, plenty of enzymes were enriched with downregulated genes under treatments I2 and I3, meaning that the normal physiological activities of *E. proxima* involved in associated enzymatic functions and metabolic pathways were hindered. In addition, the apoptosis process was also enriched with downregulated genes, which was in contrast with the previous studies [45,46,47], implying the suppression of the normal apoptosis process in *E. proxima*. Lipid droplets were shown to store intracellular excess fat [48], which were significantly enriched with upregulated genes under treatment I3; however, the promotion of mannose glucose metabolism was found under such circumstances. These results revealed that high input of inorganic fertilizer can block the lipid metabolism of *E. proxima*. In addition, a greater number of disease-related pathways in the immune system were observed to be enriched; a similar result was also observed in the study on echinoderm by Zhao [36], meaning that oxidative stress caused by inorganic fertilizer exposure might lead to disorders of function.

Under the application of inorganic fertilizer, various physiological activities of *E. proxima* were hindered, which may affect the relevant functions of *E. proxima* in the soil ecological process, such as decomposition of organic matter, nutrient cycling, maintaining the stability of soil microbiota structure, and thus disturb the soil ecological balance. In addition, because the experiment was a laboratory experiment, the food was too simple to simulate the interactions of *E. proxima* with various nutrients, microorganisms and other animals in the soil, but this experiment also excluded these disturbances and only investigated the potential impact of fertilizer application on the physiology of *E. proxima*. In the future, the experiment period can be extended to observe the changes of functional traits of collembolans under fertilizer application, and other variables can be gradually added to predict the changes of ecological functions of farmland soil in the future.

## 5. Conclusions

This study estimated the impacts of fertilizer on the transcriptome responses of collembolans. Fertilizer input resulted in an overall strong change. Fertilizer exposure duration and type were the main factors shaping the transcriptomic profiles, with relatively stable and remarkable divergent expression patterns identified after sustaining stimulation. Organic and inorganic fertilizers both impose influences in distinct ways, mainly via changing the metabolism of different molecular targets, body immunity, etc. The inorganic fertilizer effects on collembolan gene expression may be greater than the effects of organic fertilizer, which was intimately associated with input concentration. Collectively, variations in transcriptomic patterns of *E. proxima* may lead to changes in physiological and functional traits. Subtle changes in functional traits of soil animals may change the normal ecological functions by affecting their life activities such as feeding, and range of activities, and thus lead to the homeostasis of soil ecosystems. The relatively great effect of inorganic fertilizer on the collembolan is indicative of more severe disturbance and more difficult resilience of the agricultural ecosystem, in view of this, organic fertilization should be prolonged and strengthened to a reasonable extent from the aspects of agricultural sustainability and biodiversity conservation. In addition, in the future, efforts should be made to study the effects of long-term fertilizer application on the functional traits of soil animals, and the transcriptome sequencing technology should be combined to identify key genes and their interactions, so as to provide references for predicting the future functional changes of agricultural ecosystems through the changes of functional traits of soil animals.

## Figures and Tables

**Table 1:** Two-way variance analysis of variation of expression of common DEGs from pathways of glutathione metabolism and arachidonic acid metabolism under exposure duration and concentration of inorganic fertilizer.

Gene Expression	Duration				Concentration				Duration × Concentration			
	*df*	*p*	*R* ^2^	*F*	*df*	*p*	*R* ^2^	*F*	*df*	*p*	*R* ^2^	*F*
TRINITY_DN1544_c0_g1	2	˂0.001	3070.621	82.424	2	0.323	44.922	1.206	4	0.122	78.621	2.110
TRINITY_DN167_c0_g2	2	˂0.001	17.173	13.829	2	0.764	0.340	0.274	4	0.893	0.336	0.271
TRINITY_DN1979_c0_g1	2	˂0.001	220.651	35.371	2	0.283	8.458	1.356	4	0.033	20.730	3.323
TRINITY_DN30532_c0_g1	2	˂0.001	7.175	35.322	2	0.844	0.035	0.172	4	0.884	0.058	0.284
TRINITY_DN31931_c1_g1	2	˂0.001	199.562	43.887	2	0.128	10.483	2.305	4	0.189	7.830	1.722
TRINITY_DN3523_c0_g1	2	˂0.001	1347.576	44.219	2	0.388	30.447	0.999	4	0.210	49.687	1.630
TRINITY_DN13471_c0_g2	2	˂0.001	51,054.440	15.265	2	0.460	2712.385	0.811	4	0.732	1690.552	0.505
TRINITY_DN167_c0_g1	2	˂0.001	16.050	12.760	2	0.346	1.415	1.125	4	0.368	1.439	1.144
TRINITY_DN25932_c0_g1	2	˂0.001	25,901.322	101.637	2	0.165	507.931	1.993	4	0.003	1509.255	5.922
TRINITY_DN35977_c0_g1	2	˂0.001	615.572	43.848	2	0.432	12.361	0.880	4	0.068	37.124	2.644
TRINITY_DN6077_c0_g1	2	˂0.001	2224.836	37.721	2	0.879	7.662	0.130	4	0.800	24.134	0.409

**Table 2:** Two-way variance analysis of variation of CAT and SOD activities under exposure duration and concentration of inorganic fertilizer.

Enzyme Activity (U/ind.)	Duration				Concentration				Duration × Concentration			
	*df*	*p*	*R* ^2^	*F*	*df*	*p*	*R* ^2^	*F*	*df*	*p*	*R* ^2^	*F*
CAT	2	˂0.001	0.430	87.963	2	0.004	0.032	6.591	4	˂0.001	0.037	7.645
SOD	2	˂0.001	0.266	55.277	2	0.001	0.038	7.910	4	˂0.001	0.040	8.245

**Table 3:** Two-way variance analysis of variation of the number of DEGs under exposure duration and concentration of inorganic fertilizer.

Item	Duration				Concentration				Duration × Concentration			
	*df*	*p*	*R^2^*	*F*	*df*	*p*	*R^2^*	*F*	*df*	*p*	*R^2^*	*F*
**DEGs number**	2	˂0.001	1,9067,320.48	27.938	2	0.854	108,775.59	0.159	4	0.944	125,415.82	0.184

## Data Availability

No data were used for the research described in the article.
